# Achilles Tendon Tear Following an Unusual Pattern of Open Pure Ankle Dislocation: A Case Report

**DOI:** 10.7759/cureus.53814

**Published:** 2024-02-07

**Authors:** Taha El aissaoui, Aboubacar Lawan, Adnane Lachkar, Najib Abdeljaouad, Hicham Yacoubi

**Affiliations:** 1 Department of Traumatology and Orthopedics, Mohammed VI University Hospital, Faculty of Medicine and Pharmacy, Mohammed Ist University, Oujda, MAR; 2 Orthopedics, Mohammed VI University Hospital, Oujda, MAR

**Keywords:** motorcycle accident, achilles tendon repair, achilles tendon injury, open dislocation, subtalar dislocation

## Abstract

We present a rare case of concurrent open Achilles tendon tear and pure subtalar dislocation in a 20-year-old male following a motorcycle accident, highlighting the complexity of managing such unique musculoskeletal injuries. The patient was initially admitted with an open and deformed left ankle, underwent prompt primary debridement, and received immediate medical intervention. Despite unsuccessful attempts to reduce the dislocation through external maneuvers, subsequent radiographic evaluation revealed a subtalar dislocation associated with a 90° rotation of the talus.

A direct reduction was achieved in the operating room, uncovering a complete tear of the Achilles tendon and a section of the posterior tibial artery during more intensive debridement. The vascular surgery team repaired the posterior tibial artery, followed by Achilles tendon repair using a Kessler suture technique. Postoperative care involved immobilization and adapted rehabilitation, resulting in the patient regaining full range of motion without complications.

To the best of our knowledge, this case represents the first reported open lateral subtalar dislocation associated with an open Achilles tendon tear. The detailed treatment strategies and outcomes offer valuable insights for clinicians facing similar challenges and inspire further research on rare musculoskeletal injuries.

## Introduction

In contrast to closed Achilles tendon injuries, open Achilles tendon tears are infrequent, as highlighted by their rarity in the literature [1]. The limited available literature underscores the scarcity of information on this type of injury [2].

Subtalar dislocations constitute another rare entity, accounting for only approximately 1% of all traumatic dislocations [3]. Typically resulting from high-energy injuries like falls from a height or motor vehicle accidents [4], these dislocations are open in 10 to 40% of cases [5], with pure forms being exceptionally rare [6].

The coexistence of these conditions presents a highly challenging scenario. This paper details an exceptionally unique case involving the confluence of two rare injuries: an open Achilles tendon tear and an open pure subtalar dislocation, associated with a posterior tibial artery section in a 20-year-old male following a motorcycle accident.

## Case presentation

**Figure 1:**
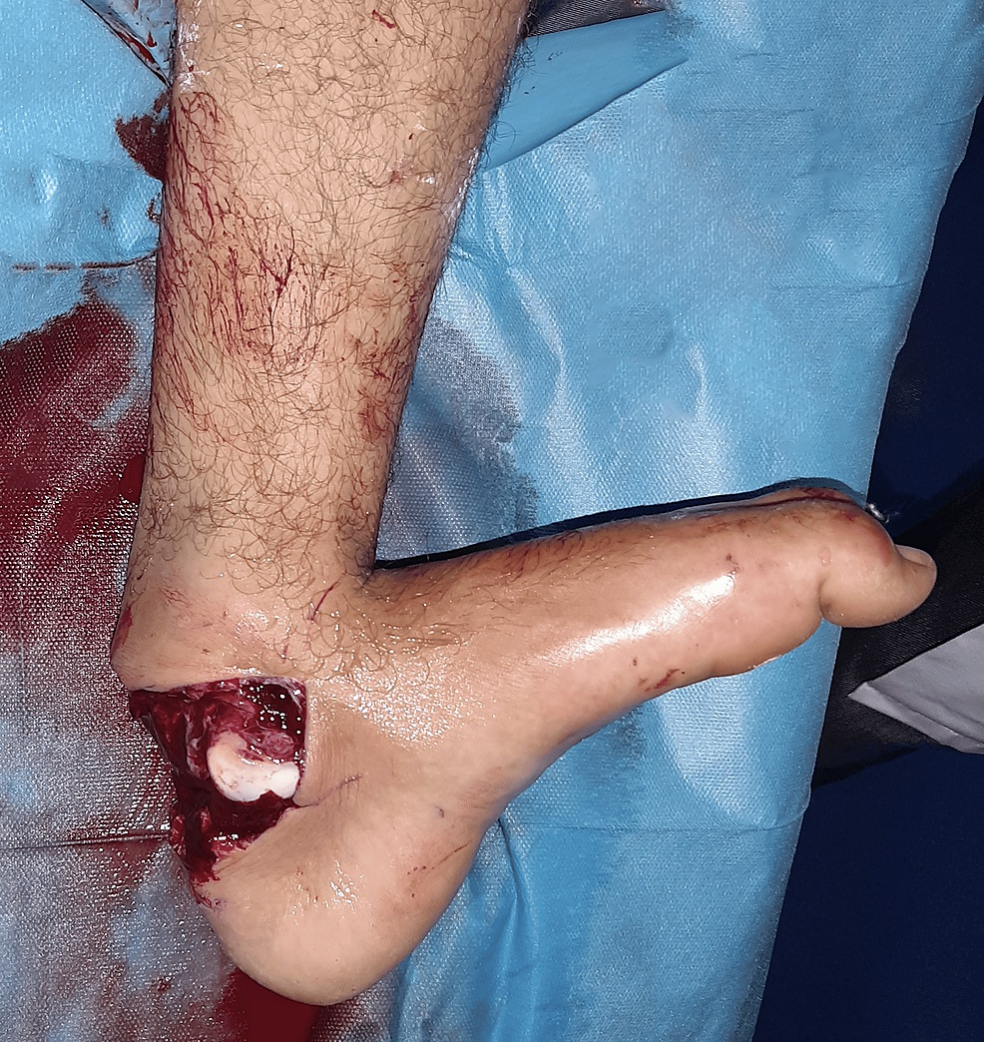
Clinical image of the injured ankle.

We present the case of a previously healthy 20-year-old male admitted to our department following a motorcycle accident resulting in an open injury to his left ankle. Upon initial examination, we observed an open and deformed left ankle with an 8cm posterior skin wound (Figure 1). The posterior tibialis artery pulse was not palpable, while the dorsalis pedis pulse was present. Due to the extreme pain expressed by the patient, a comprehensive neurological evaluation was limited.

**Figure 2:**
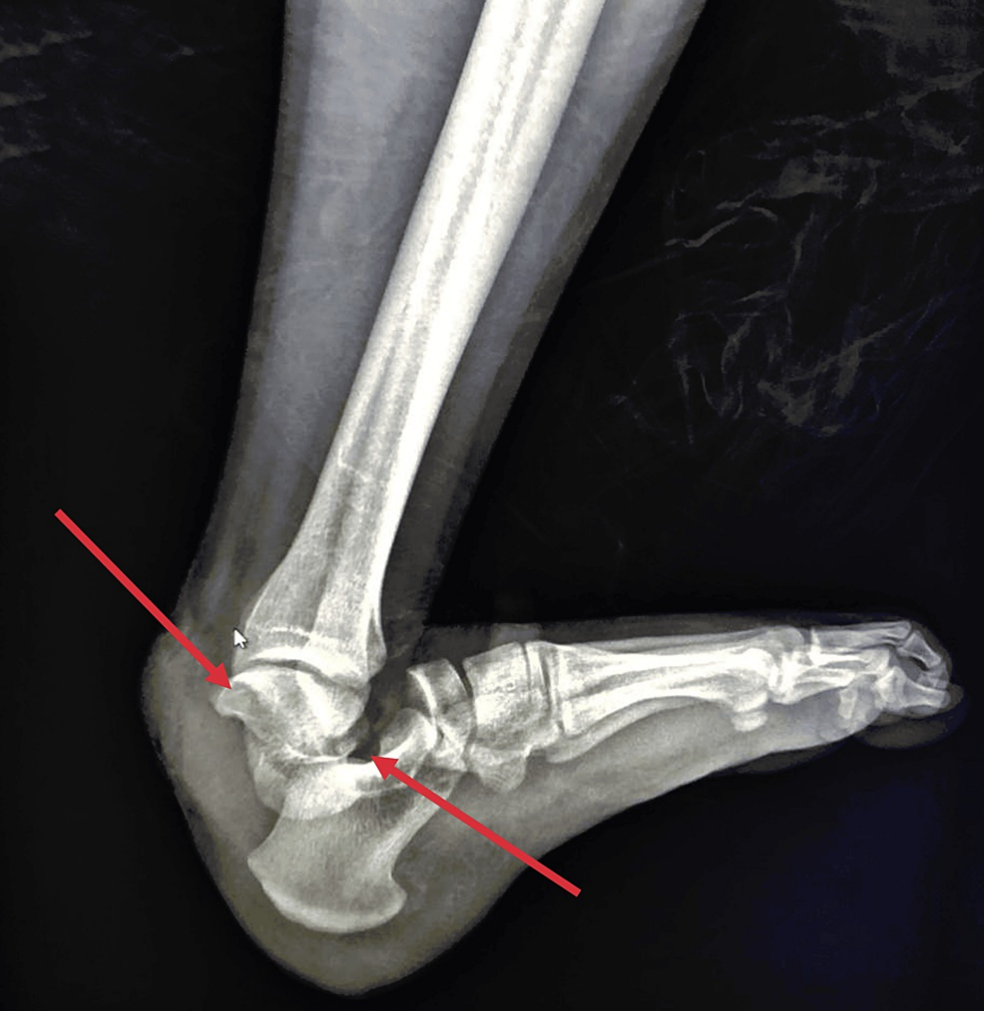
Lateral radiographic view of the left ankle. The arrows designate the subtalar dislocation and the medial rotation of the talus.

**Figure 3:**
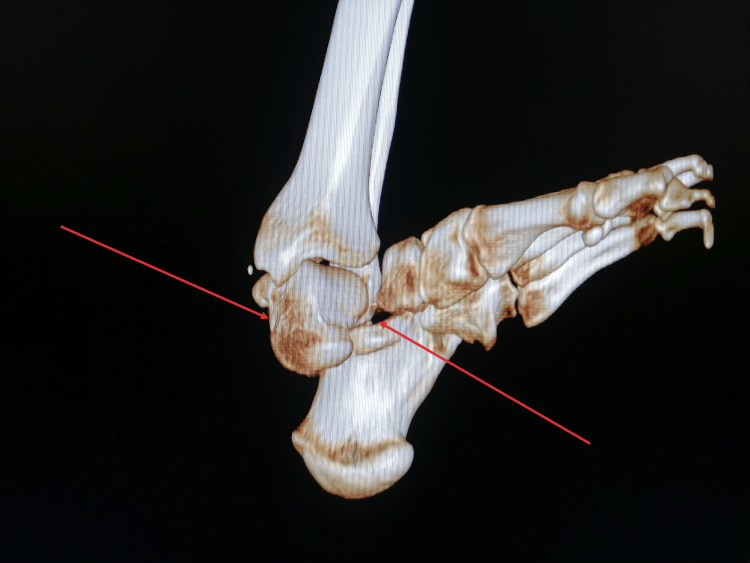
3D CT-scan reconstruction of the left ankle. The arrows designate the subtalar dislocation and the medial rotation of the talus.

**Figure 4:**
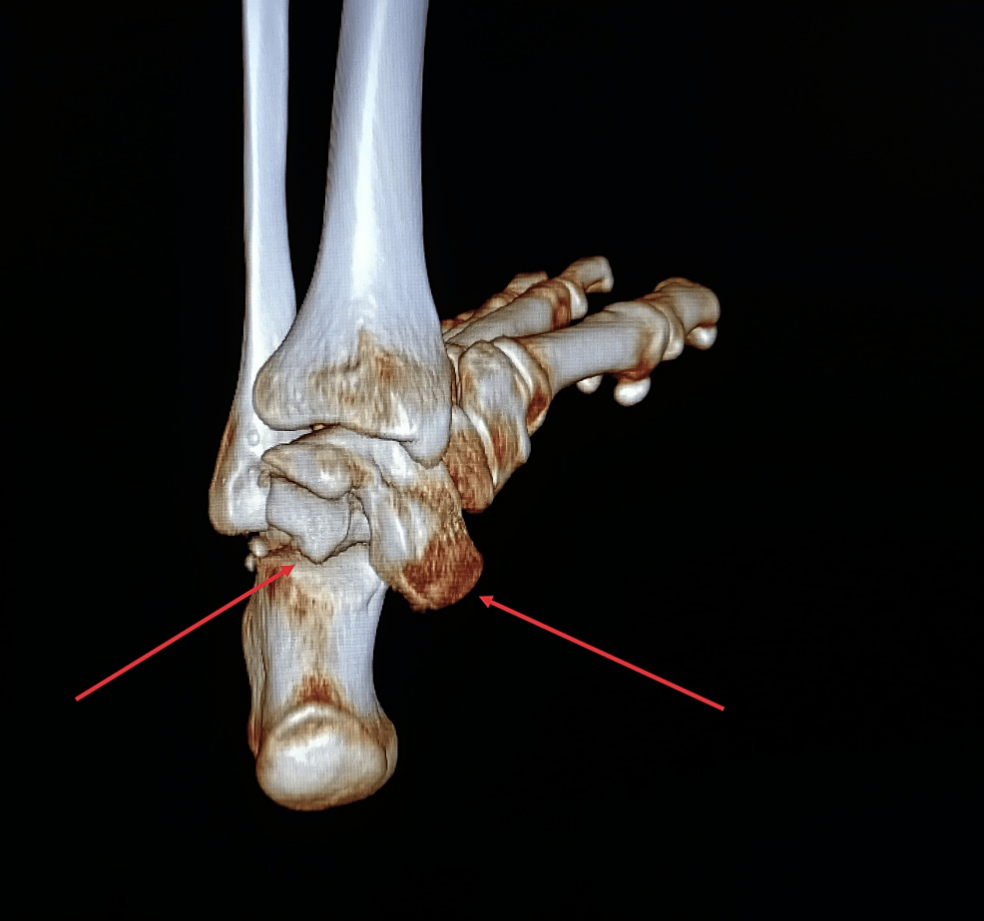
3D CT-scan reconstruction of the left ankle. The arrows designate the subtalar dislocation and the medial rotation of the talus.

Following prompt primary debridement, the patient received immediate administration of one gram of amoxicillin/clavulanic acid, 160 mg of gentamicin, a tetanus vaccine, and tetanus antitoxin. Attempts to reduce the dislocation through external maneuvers under sedation were unsuccessful. Subsequent radiographic evaluation and a CT scan revealed a subtalar dislocation associated with a 90° rotation of the talus (Figure 2-4). The patient was immediately transferred to the operating room, where the direct reduction was achieved using a small Hohmann retractor to reposition the talus. Subsequently, longitudinal traction was applied to the calcaneus to reduce the subtalar dislocation.

**Figure 5:**
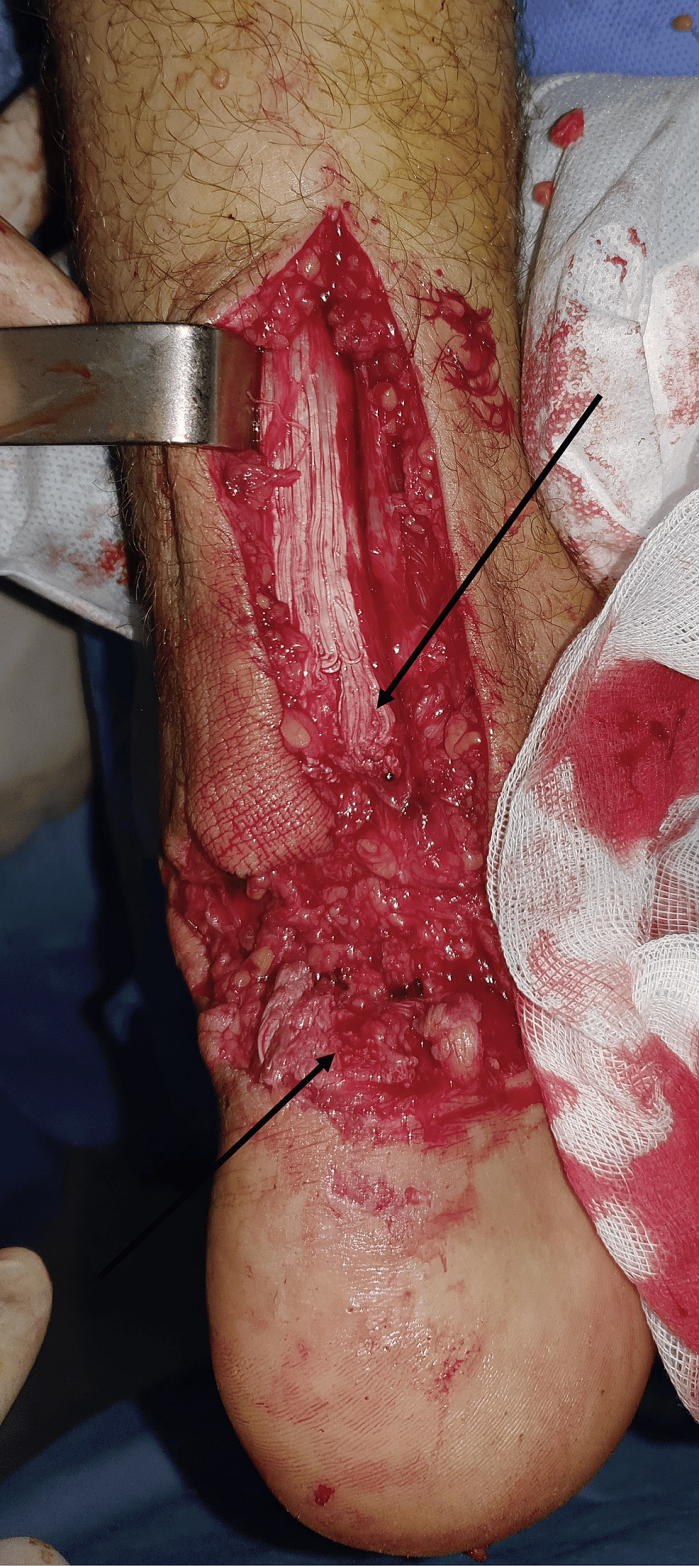
Intraoperative images of Achilles tendon tear. The arrows in the intraoperative image designate the proximal and distal ends of the Achilles tendon.

**Figure 6:**
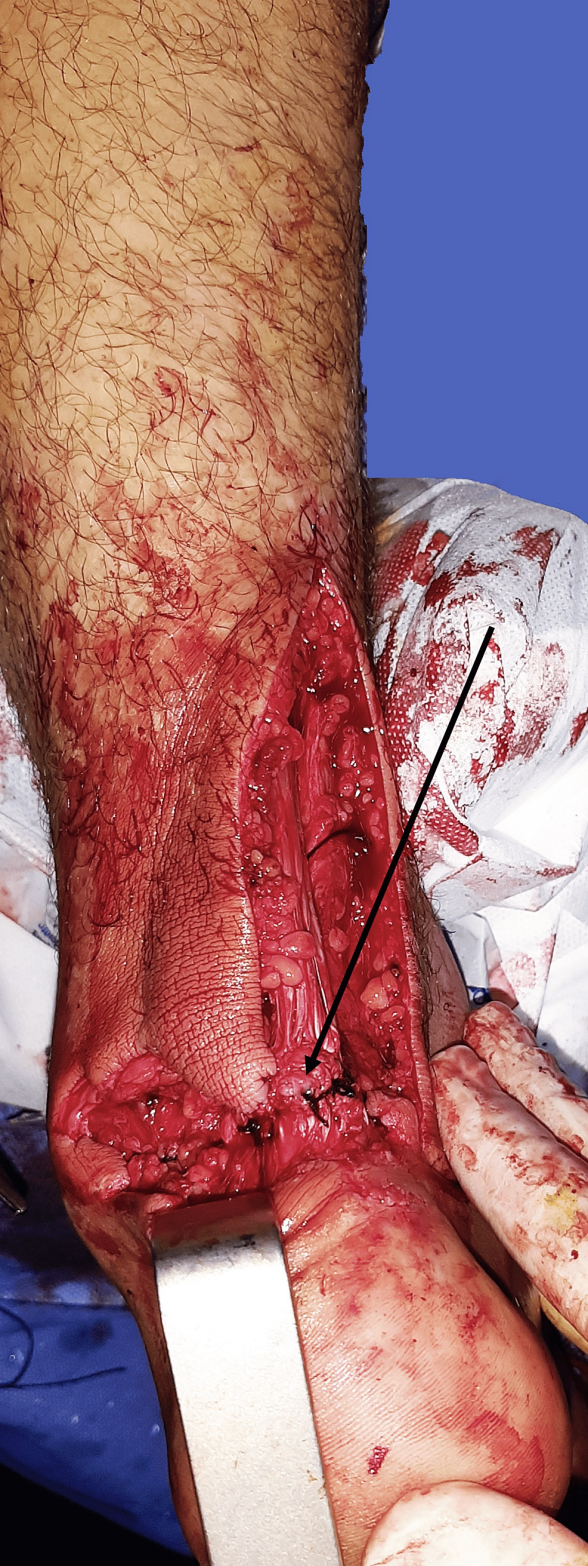
Intraoperative images of Achilles tendon repair. The arrow designates the level of the Kessler suture and the restoration of the Achilles tendon continuity.

**Figure 7:**
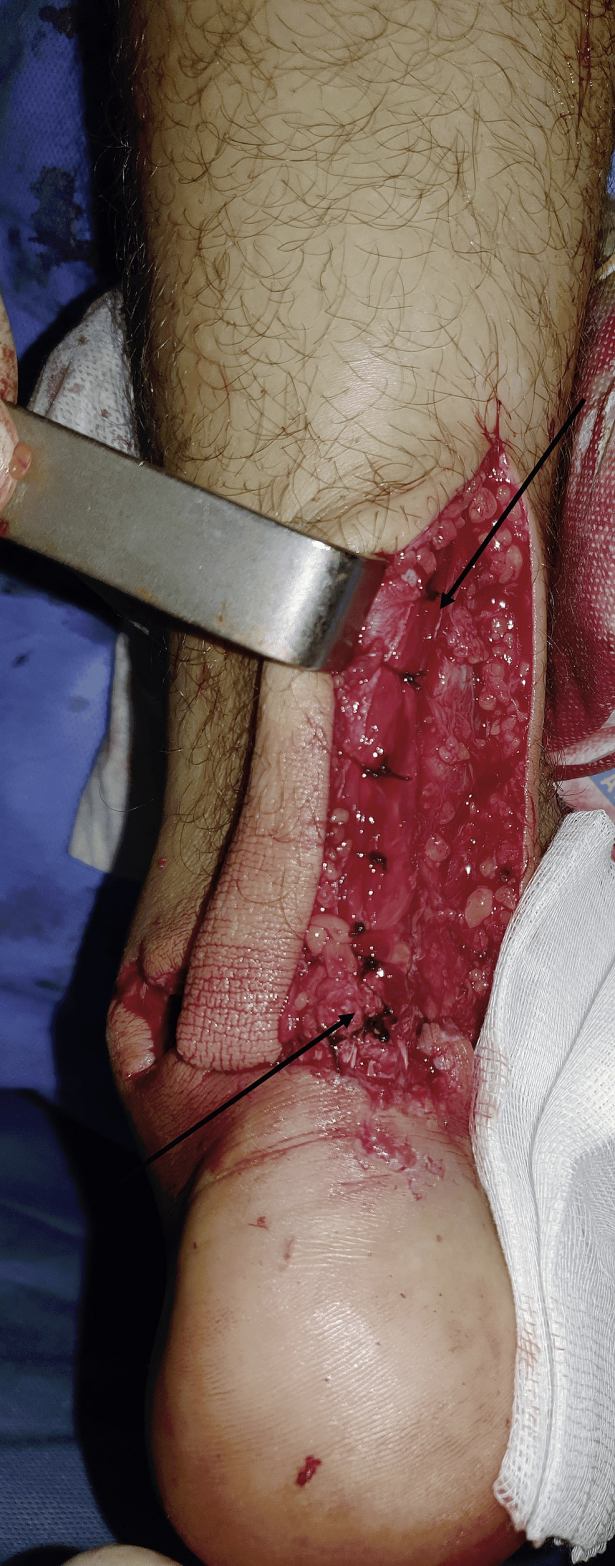
Intraoperative images of the paratenon repair. The arrows designate the closure of the paratenon using simple sutures.

Upon successful ankle reduction, we proceeded with more intensive debridement and meticulous injury assessment, revealing a complete tear of the Achilles tendon and section of the posterior tibial artery (Figure 5). No further lesions or foreign bodies were found. The vascular surgery team repaired the posterior tibial artery by terminal suture; then, we repaired the Achilles tendon using a Kessler suture and closed the paratenon (Figure 6,7).

The patient underwent a five-day course of intravenous amoxicillin/clavulanic acid at a dose of 1 g three times per day, along with gentamicin for two days at a dose of 160 mg per day. Additionally, the patient received enoxaparin sodium at a dose of 4,000 IU per day for six weeks. Immobilization was achieved using a short leg cast in full flexion for three weeks, followed by three weeks in a neutral position. Weight-bearing, passive, and active ankle mobilization were permitted starting in the seventh week following an adapted rehabilitation program. After three years of follow-up, the patient regained full range of motion without complications.

## Discussion

In 1803, Hey [7] was the first to describe subtalar dislocation, an infrequent injury classified for the first time by Broca [8] in 1852, who distinguished subtalar dislocations from complete talus enucleation. Lateral dislocation typically results from forced eversion, a blocked foot, and lateral force applied to the extremity [9]. This injury may be associated with skin disruption, leading to open dislocation in 22% of cases [10].

Up to 20% of lateral ankle dislocations may be irreducible due to osseous or tendinous interposition [11]. Anatomical structures, such as the navicular or the posterior tibialis and the flexor hallucis longus, tend to engage and prevent an effective reduction maneuver.

Surgical interventions, including debridement, repair of associated lesions, and ankle coverage in cases of significant skin loss, are deemed necessary [12]. Various stabilization methods, such as K-wires [13], short leg casts [14], and external fixators [14], are employed. Immobilization is continued for six weeks, followed by weight-bearing and ankle rehabilitation [15].

Complications predominantly include stiffness and osteoarthritis at the subtalar joint [16]. Flat foot results from inadequate healing of ligamentous structures, leading to a collapse of the internal arch during loading [15]. The risk of talus necrosis is low because its vascularization is preserved in dislocation, unlike in fracture-dislocation of the talus [13].

Only a few reports on open Achilles tendon injuries are available in the literature [17]. To the best of our knowledge, the present case represents the first report of an open lateral subtalar dislocation associated with an open Achilles tendon tear.

Due to the risk of infection and the compromised soft tissue envelope, Achilles tendon lacerations are often challenging. In our case, after the dislocation reduction, we extended the skin incision proximally over the medial border of the tendon. We proceeded with thorough debridement, followed by regularization of the tendon ends. We repaired the tendon using Kessler sutures. Finally, the surrounding paratenon was restored with 2-0 VICRYL before the wound was closed.

Huang et al. [18] and Brumann et al. [19] recommended full weight bearing immediately after repair with early ankle joint range of motion exercises. Given the unusual injury combination and the seriousness of the mechanism, we opted for immobilization for three weeks in plantar flexion, followed by three weeks in a neutral position with no weight bearing.

Our patient presented good results with no range of motion restriction and no sign of infection, skin necrosis, or degenerative complications.

## Conclusions

In conclusion, our paper presents a distinctive case of concurrent open Achilles tendon tear and pure subtalar dislocation, emphasizing the complexity of managing rare musculoskeletal injuries. The successful surgical interventions and three-year follow-up underscore the importance of tailored approaches in achieving positive outcomes.

This case contributes to the limited literature on open Achilles tendon injuries, marking the first reported instance of an open lateral subtalar dislocation associated with such a tear and posterior tibial artery section. The discussion on treatment strategies, including innovative surgical techniques, provides valuable insights for future clinical considerations. In summary, our case report addresses the challenges of rare musculoskeletal injuries and adds to the existing literature, offering practical information for clinicians and inspiring further research in this field.
